# 

**Published:** 1892-12

**Authors:** 


### CONTENTS:

| | | PAGE |
|---|---|---|
| ORIGINAL ARTICLES: | | |
| | Rheumatism in Horses. By T. V. Hinebauch, M. S., V. S. | 725 |
| | Sclerostoma Tetracanthum. By G. L. Buffington, D. V. M. | 734 |
| | Report of Committee on Sanitary Science and Police of Pennsylvania State Medical Association | 742 |
| | Report of Committee on Diseases. By A. W. Clement, V. S. | 749 |
| | Coal Oil Poisoning. By W. H. Ridge, V. M. D. | 757 |
| COMMUNICATION: | | |
| | Tuberculosis. By John M. Parker, D. V. S. | 759 |
| EDITORIAL: | | |
| | New York State Veterinary Medical Society | 761 |
| | Resolutions of Respect | 761 |
| | Tuberculosis | 762 |
| SOCIETY PROCEEDINGS: | | |
| | Proceedings of the Association for the Study of Comparative Psychology of McGill University, Montreal | 765 |
| | California State Veterinary Medical Association | 776 |
| | Massachusetts Veterinary Association | 776 |
| | Iowa State Veterinary Medical Association | 780 |
| | Philadelphia Veterinary Medical Association | 781 |
| | United States Veterinary Medical Association—Officers and Committees, 1892–3 | 786 |
| Index | | 787 |

